# Intracranial Hypertension and Optic Neuritis in Two Unrelated Seronegative Cases

**DOI:** 10.1002/ccr3.70041

**Published:** 2024-12-31

**Authors:** Reza Nejad Shahrokh Abadi, Farid Shekarchian, Farrokh Seilanian Toosi, Ahmadreza Mashreghi, Sara Maddahpour, Samane Kamali, Shima Shekari, Mehran Beiraghi Toosi, Narges Hashemi

**Affiliations:** ^1^ Clinical Research Development Unit, Ghaem Hospital, Faculty of Medicine Mashhad University of Medical Sciences Mashhad Iran; ^2^ Eye Research Center Mashhad University of Medical Sciences Mashhad Iran; ^3^ Department of Radiology, School of Medicine Mashhad University of Medical Sciences Mashhad Iran; ^4^ Mashhad University of Medical Sciences Mashhad Iran; ^5^ Department of Pediatrics, School of Medicine Mashhad University of Medical Sciences Mashhad Iran

**Keywords:** ICHT, optic neuritis, pediatric neurology, seronegativity

## Abstract

Intracranial Hypertension (ICHT) is identified as the elevation of Cerebrospinal Fluid (CSF) pressure in patients devoid of any underlying causes. Optic Neuritis (ON) is not typically seen as a complication of ICHT, and patients diagnosed with concurrent manifestation of both these disorders usually have some identifiable underlying cause. In this report, we highlight the clinical and para‐clinical findings in two unrelated children presenting with high CSF opening pressures and Optic neuritis in the absence of any identifiable neurological or immunological cause.

**Summary**

- Patients presenting with Optic Neuritis (ON) may have associated Intracranial Hypertension, even with normal body mass index (BMI).
- This concurrent manifestation may be in the absence of any other cause, including vasculitis, anti‐Myelin Oligodendrocyte Glycoprotein IgG (MOG) and anti‐Aquaporin4 (AQP4) antibody, and Multiple Sclerosis (MS).

## 1 Introduction

Intracranial Hypertension (ICHT) is the elevation in Cerebrospinal Fluid (CSF) pressure in the absence of any discernible neurological pathology such as lesions or ventricular dilatation [1]. ICHT predominantly impacts young and overweight women, and may be accompanied by other symptoms such as headaches, pulsatile tinnitus, and visual disturbances or diplopia in the majority of cases [2, 3]. Papilledema represents the most severe clinical presentation of ICHT and may be accompanied by varying degrees of ophthalgia [4]. The pathophysiology of papilledema is thought to occur as a result of impaired axoplasmic flow, secondary to increased Intracranial Pressure (ICP), which in the long term can lead to optic nerve damage [1]. However, Optic Neuritis (ON), which presents as acute or subacute vision loss accompanied by periorbital pain, dyschromatopsia, and visual field defects [5], is not a complication of ICHT and the coexistence of these two conditions should raise suspicion for another underlying disease. This case report discusses the clinical course, diagnostic measures, and treatment of two unrelated adolescent girls with concurrent occurrence of ICHT and ON, and explores the potential causes of this co‐occurrence.

## 2 Case History/Examination

### 2.1 Case 1

**FIGURE 1:**
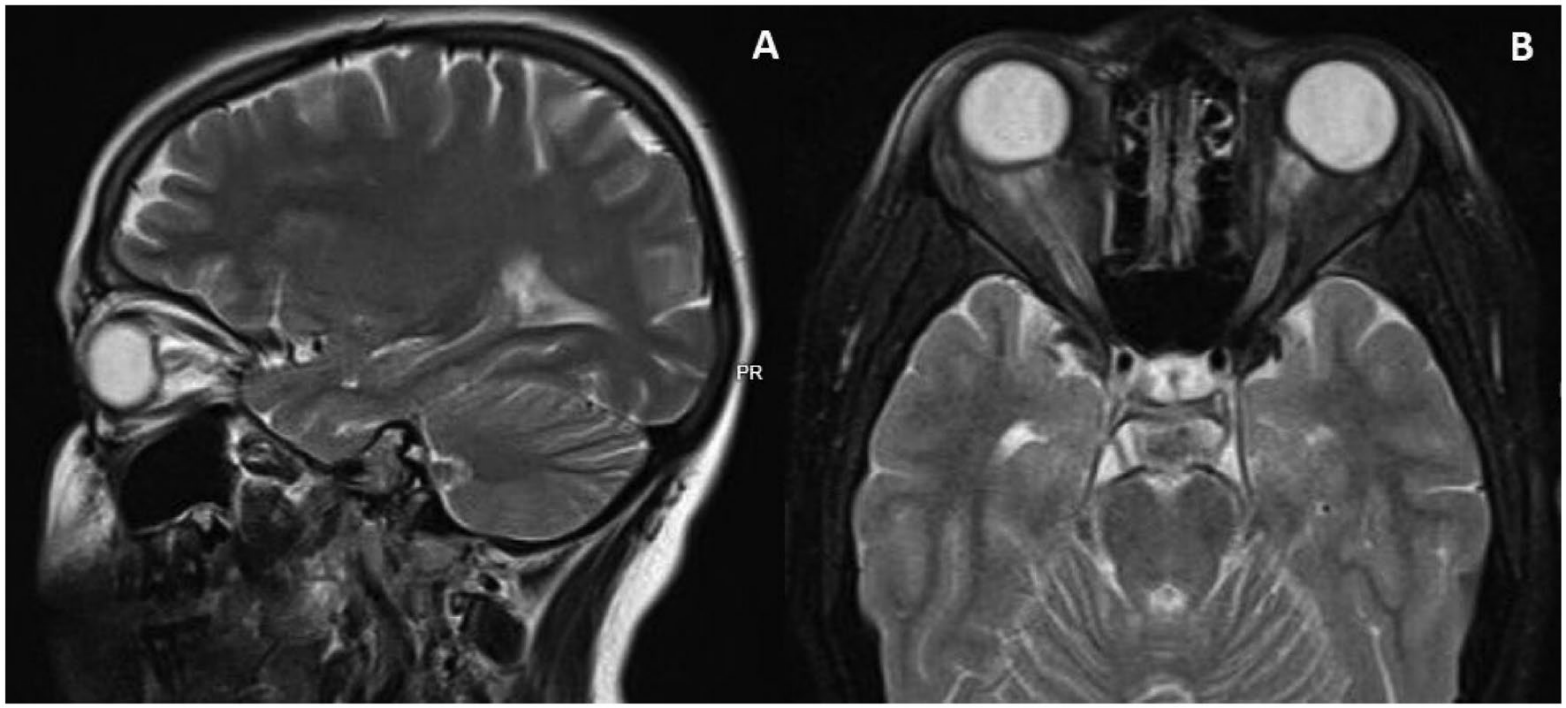
Brain MRI of Case 1 during her first admission. (A) Sagittal MRI showing widening of Optic Nerve subarachnoid space. (B) Axial MRI showing flattening of posterior aspect of the globe.

A 15‐year‐old girl, born to non‐consanguineous parents, without any history of past neurological disorders or neurodevelopmental delay (BMI of 18.3). Her parents report tension‐type headaches along with low‐grade fever, nausea, and vomiting a week before admission. On the day of admission, she experienced sudden bilateral visual loss (left dominant) as well as faint tinnitus. Her headaches also worsened and developed a pulsatile quality that worsened with Valsalva maneuvers. She was not on any significant medications, nor did she have a history of chronic supratherapeutic Vitamin‐D supplementation. On examination, she had optic nerve swelling, bilateral papilledema, and optic disc pallor. All other neurological assessments, including muscle force, hearing, cranial nerves, DTRs, and sensorium were normal. Laboratory results and lumbar puncture (LP) are detailed in Table 1, and CSF opening pressure was documented as an elevated 52 cmH2O. Brain MRI showed bilateral bulging of the optic discs, with no pathological findings in contrast enhancement, while Orbital MRI showed signs of bilateral Optic Nerve sheath dilation with tortuosity, along with concavity in optic discs (Figure 1). Cervical and Thoracic MRIs were normal, brain MRV showed hypoplastic left Transverse Sinus without signs of thrombosis. An initial diagnosis of ON along with ICHT was made and the patient was started on Methylprednisolone and IVIg and discharged after 22 days with Prednisolone, Topiramate, and Acetazolamide.

**TABLE 1:** Laboratory values of both patients.

Value	Case 1 (1st)	Case 1 (2nd)	Case 2	Unit	Normal range
WBC	8.5	8.4	13.3	10^3^/µL	4–10
PMN	73.7	52.9	67.2	%	30–70
Lymphocytes	18	33.4	24.9	%	2050
Platelets	144	165	255	10^3^/µ	150–450
Hemoglobin	12.1	13.7	12.2	g/dL	13–17
ESR	13	14	17	mm	< 10
CRP	5.9	5	4.3	mg/L	< 6
Sodium	138	—	141	mEq/L	135–145
Potassium	4.1	—	3.9	mEq/L	3.5–5.5
Creatinine	0.8	—	0.7	mg/dL	0.6–1.1
Magnesium	2.0	—	1.7	mg/dL	1.6–2.6
Phosphor	4.3	—	3.2	mg/dL	2.8–4.5
Calcium	8.3	7.7	10.2	mg/L	8.5–10.5
CSF Pressure	52	30	47.5	cmH2O	< 25
CSF WBC	0	0	0	/mm^3^	—
CSF Sugar	58	62	126	mg/dL	45–85
CSF Protein	27	63.8	193	mg/dL	15–45
CSF LDH	52	35	27	U/L	< 40
p‐ANCA	—	5.4	6.7	U/mL	Positive > 20
c‐ANCA	—	10	8	U/mL	Positive > 20
ANA	—	0.8	2.1	U/mL	Positive > 55
Anti‐ds DNA	—	5.4	10.9	U/mL	Positive > 40

Abbreviations: ANA, antinuclear antibody; ANCA, anti‐neutrophil cytoplasmic antibody; CRP, C‐reactive protein; CSF, cerebrospinal fluid; ESR, erythrocyte sedimentation rate; LDH, lactate dehydrogenase; PMN, polymorphonuclear; WBC, white blood cells.

### 2.2 Case 2

A 13‐year old girl, with any significant past medical history (BMI of 17.5), born to non‐consanguineous parents, reported rhinitis and low‐grade fever a week prior to admission. The patient had a history of worsening Occipital headaches, starting from a month prior, each lasting for about 4 h and repeating several times throughout the day. Similar to Case 1, she experienced sudden onset bilateral vision loss (left dominant), tinnitus, and headaches, which worsened with Valsalva maneuvers, on admission. On examination, on top of bilateral papilledema, she had slightly reduced upper distal muscle force (grade 4 out of 5) and paresthesia. Similar to Case 1, she had no significant drug history, and denied past history of similar symptoms. Laboratory results and LP are detailed in Table 1, and CSF opening pressure was documented as an elevated 47.5 cmH2O. Brain, Orbital, and Cervicothoracic MRIs were all normal, and devoid of any pathological findings or contrast enhancements. Subsequently, she was diagnosed with bilateral ON and ICHT, and started on Methylprednisolone and IVIg, and discharged with Acetazolamide and Prednisolone after 10 days.

## 3 Methods (Differential Diagnosis, Investigations, and Treatment)

CSF cultures and HSV PCRs were negative and lacked any atypical cells, additionally, nasopharyngeal and oral PCR swabs for SARS‐COV‐2 and Influenza typing were negative for both patients. Visual Evoked Potential (VEP) in both patients shows signs of bilateral latency in the pre‐chiasmatic visual pathways. Both patients were evaluated for several rheumatological and neurological conditions on discharge in light of the concurrent manifestation of ON and ICHT. Vasculitis markers, p‐ANCA and c‐ANCA, were negative in both patients (Table 1). In addition, anti‐Myelin Oligodendrocyte Glycoprotein IgG (MOG) and anti‐Aquaporin4 (AQP4) antibody were normal in both patients. Neither one of the patients had signs indicative of Multiple Sclerosis (MS) plaques in follow‐up MRIs. All rheumatological markers, including ANA, RF, anti‐CCP, Jo‐1, and anti‐dsDNA, were within normal limits for both patients (Table 1). Moreover, optic nerve head, retinal nerve fiber layer, and macular Ganglion Cell Complex (GCC) Optical Coherence Tomography (OCT) imaging revealed true bilateral optic nerve elevation in Case 1 and bilateral optic atrophy in Case 2.

## 4 Conclusion and Results (Outcome and Follow‐Up)

Regarding Case 1, 40 days after discharge, the patient stopped taking all her prescribed medications, and returned after 2 weeks with the same symptoms in addition to ophthalmalgia. This time the CSF opening pressure was 30 cmH2O, and all neuro‐imaging studies were normal. Similar treatment was once again initiated and the patient was discharged after 4 days. The patient was followed‐up after 2 months, wherein her sight had returned to normal, although she still complained of recurring left eye pain and mild headaches. Case 2 was followed‐up after 3 months, she was myopic in both eyes and had a visual acuity of 2/6 along with mild horizontal diplopia.

## 5 Discussion

This report describes two young girls who presented with initial symptoms of headache and sudden onset bilateral vision loss, and were ultimately diagnosed with ICHT and ON. Sudden bilateral vision loss in these two patients prompted consideration of several potential differential diagnoses in addition to ON, including: Nonarteritic Anterior Ischemic Optic Neuropathy (NAION), Papilledema with rapidly progressive vision loss due to space‐occupying CNS lesion, Cerebral Sinus Venous Thrombosis (CVST), Fulminant IIH, Trauma, Intoxication, and Basilar Migraine [6]. Based on the parents' history and the results of U/A toxicology, trauma and intoxication were ruled out. Moreover, MRV, Orbital and Brain MRI performed on the patients revealed bilateral bulging of the Optic Discs and bilateral Optic Nerve sheath dilation, which is diagnostic for IIH [7]. Furthermore, no evidence of thrombosis or distinct CNS lesions was seen in MRI and MRV, ruling out space‐occupying pathologies and CVST. Following these evaluations, a diagnosis of ICHT was made, and to rule‐out fulminant IIH as the potential cause, VEP testing was conducted, which reported latency in both patients' pre‐chiasmatic pathways, ultimately leading to the diagnosis of ICHT accompanied by bilateral ON.

Since ON is not known as a complication of ICHT, the coexistence of these two conditions in a patient should raise suspicion of another underlying disease. Sorensen [8] and Maran et al. [9], reported two patients with concurrent ON and ICHT, but were ultimately diagnosed with Myelin Oligodendrocyte Glycoprotein‐Associated Disease (MOGAD), which was tested for and found to be negative in both patients. Additionally, Srimanan et al. reported the coexistence of ICHT and ON in a patient with Systemic Lupus Erythematosus (SLE) [10], which was ruled out in these patients. Furthermore, Sardar et al. reported the simultaneous occurrence of these two conditions in an individual with a history of COVID‐19 infection, which was not detected via PCR in either patient [11].

Due to the OCT findings conclusive for optic nerve elevation and atrophy in the patients, coupled with documented papilledema in the physical examination and relatively sudden bilateral visual loss, two distinct differential diagnosis come to mind. First, Acute disseminated encephalomyelitis (ADEM), which was ruled out in the initial and follow‐up MRIs. Second, Neuromyelitis Optica Spectrum Disorder (NMOSD). The diagnosis of NMOSD in the setting of negative or unknown AQP4 antibodies requires two core clinical characteristics with dissemination in space and accompanying MRI findings. Both of these patients had the core clinical finding of ON, along with normal findings in the MRI. However, no other core clinical characteristic could be identified; including acute myelitis, area postrema syndrome, or brainstem syndrome; moreover, none of the patients met the dissemination in space criteria.

Interestingly, both patients had elevated levels protein found in their CSF (Table 1). Some differential diagnosis of simultaneously increased ICP and elevated CSF protein include, and previously ruled out, MS, CVST, vasculitic disorders, SLE, NMOSD, and viral or bacterial CNS infections. The documented cyto‐albuminologic dissociation seen in these patients is typical of that of Guillain–Barré syndrome (GBS), which could rarely be accompanied with ICHT and ON, although other typical diagnostic features were not seen in these patients [12, 13]. Neuro‐sarcoidosis is another condition associated with increased CSF protein and benign ICHT, with optic nerve involvement seldom seen [14, 15]. However, these patients had no past history of sarcoidosis, nor had they any of the typical findings. Furthermore, no findings suggestive of neoplastic disorders (i.e., lymphoma or leukemia) were noted in either patient. One possible explanation for these symptoms, particularly in Case 2, is potentially Chronic Inflammatory Demyelinating Polyradiculoneuropathy (CIDP), which is rarely associated with ICHT and ON, and can cause an isolated increase in CSF proteins [16, 17]. However, CIDP is incredibly rare in children, with an approximate prevalence of 0.22 per 100,000 children, and additionally and in particular Case 1, our patients failed to fulfill the required diagnostic criteria of CIDP [18].

In the evaluation of ON in these two pediatric cases, a comprehensive assessment was conducted, encompassing the exclusion of genetic, infectious, and neoplastic etiologies [5]. Serological testing for anti‐Myelin MOG and AQP4 antibodies yielded results within normal limits in both patients. Moreover, the patients failed to fulfill the required diagnostic criteria for NMOSD. Rheumatologic investigations were also undertaken to explore potential associated comorbidities, all of which demonstrated values within the reference range. Notably, clinical manifestations indicative of rheumatologic disorders was not observed in either patient. Both patients exhibited an absence of MRI‐detectable plaques, a characteristic feature of MS. Additionally, neither case had any findings indicative of CIDP, Neuro‐Sarcoidosis, or GBS. Both patients are currently under observation for long‐term follow‐up for early detection of potential underlying causes, and to assess whether the diagnosis of Chronic Relapsing Inflammatory Optic Neuritis (CRION) would be noted [5]. Ultimately, these two cases failed to fulfill the required criteria for various neurological disorders, as such a final diagnosis of concurrently manifesting ON and ICHT was concluded on.

## 6 Conclusion

The simultaneous presence of ICHT and ON (ON) in children poses a notable difficulty in diagnosis, especially when the cause is unknown. Past research has recorded instances where simultaneous ICHT and ON were linked to recognizable conditions like myelin oligodendrocyte glycoprotein antibody disease (MOGAD), SLE, COVID‐19 infection, and NMOSD. These results emphasize the significance of comprehensive diagnostic assessments because many patients with both of these symptoms usually have an underlying disorder that can guide their treatment. Our case report differs from previous studies as it showcases two patients with ICHT and ON without any known underlying disorder. This finding is significant as it points out the possibility of these two different neurological conditions occurring spontaneously. The lack of identifiable reasons in our patients contradicts the common belief that both IIH and ON always have specific underlying causes. This situation underscores the importance of thoroughly evaluating all potential causes, such as infectious, autoimmune, and metabolic factors. Even though thorough examination is important, our research shows that unfavorable outcomes may still happen. This is the first known report of patients showing ON and ICHT without a clear cause, which adds to the overall understanding of these disorders. Further exploration is needed to understand the pathophysiological mechanisms behind idiopathic cases of ICHT and ON based on these findings. Future studies should concentrate on determining possible genetic, environmental, and immunological aspects that may play a role in these idiopathic instances. Gaining a more comprehensive knowledge of these mechanisms could not only improve our ability to diagnose but also help in creating specific treatment plans for those affected.

## Author Contributions

**Reza Nejad Shahrokh Abadi:** data curation, investigation, writing – original draft, writing – review and editing. **Farid Shekarchian:** data curation, investigation. **Farrokh Seilanian Toosi:** conceptualization, validation. **Ahmadreza Mashreghi:** writing – original draft, writing – review and editing. **Sara Maddahpour:** writing – original draft, writing – review and editing. **Samane Kamali:** data curation, writing – review and editing. **Shima Shekari:** data curation, writing – review and editing. **Mehran Beiraghi Toosi:** writing – original draft, writing – review and editing. **Narges Hashemi:** conceptualization, data curation, investigation, methodology, supervision, validation.

## Acknownledgments

Data for this report was extracted from the ongoing Pediatric Acquired Demyelinating Syndromes Registry (PADSR), under the supervision of Dr. Nejad Shahrokh Abadi and Dr. Hashemi. The authors would like to appreciate the Clinical Research Development Unit, Ghaem Hospital, Mashhad University of Medical Sciences, for their assistance. This study was approved by the ethics committee of Mashhad University of Medical Sciences (MUMS), and is in compliance with all necessary protocols, guidelines, requirements, and in accordance with the ethical standards as laid down in the 1964 Declaration of Helsinki (ethics ID: IR.MUMS.REC.1402.232).

## Consent

Written informed consent for the publication of this report was obtained from the patients by the corresponding author.

## Conflicts of Interest

The authors declare no conflicts of interest.

## Data Availability

All data supporting the findings of this study are available within the paper.
